# Supplementary material for: Crystal structure and site-directed mutagenesis of circular bacteriocin plantacyclin B21AG reveals cationic and aromatic residues important for antimicrobial activity
Source: Sci Rep. 2020 Oct 15;10:17398. doi: 10.1038/s41598-020-74332-1 (PMC7562740; doi:10.1038/s41598-020-74332-1)
Supplement: Supplementary file 1 — Supplementary Information. [file 41598_2020_74332_MOESM1_ESM.pdf]

Crystal structure and site-directed mutagenesis of circular bacteriocin plantacyclin B21AG reveals cationic and aromatic residues important for antimicrobial activity

Mian-Chee Gor[1,2,+], Ben Vezina[1,+], Róisín M. McMahon[1], Gordon J. King[3], Santosh Panjikar[4,5], Bernd H. A. Rehm[1,6], Jennifer L. Martin[1,7], Andrew T. Smith[1,8,*]

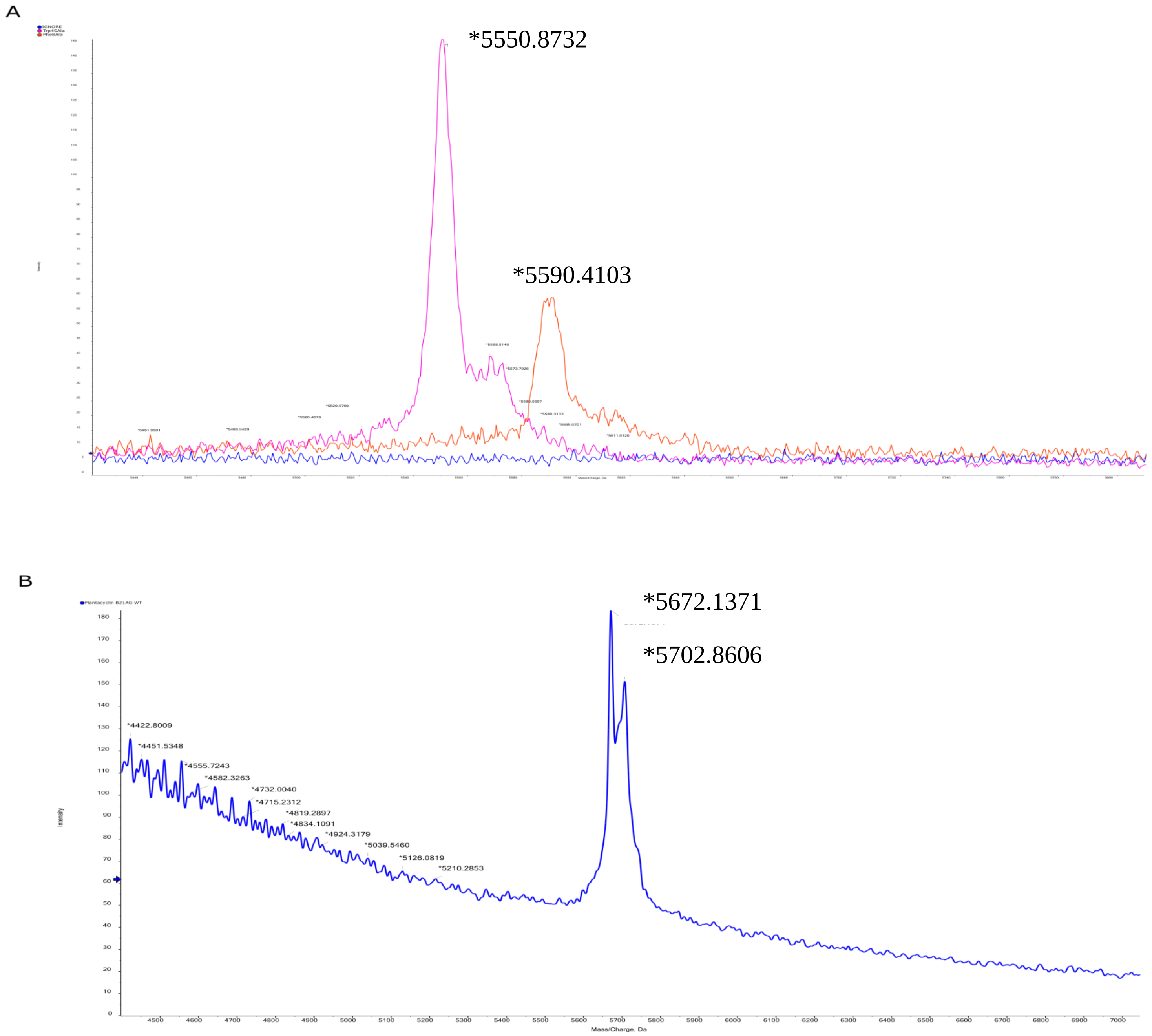

Figure S1. MALDI-TOF traces of bacteriocin and mutants. **A** shows plantacyclin B21AG Trp^{45}Ala in pink and plantacyclin B21AG Phe^{8}Ala in orange. **B** shows plantacyclin B21AG WT.

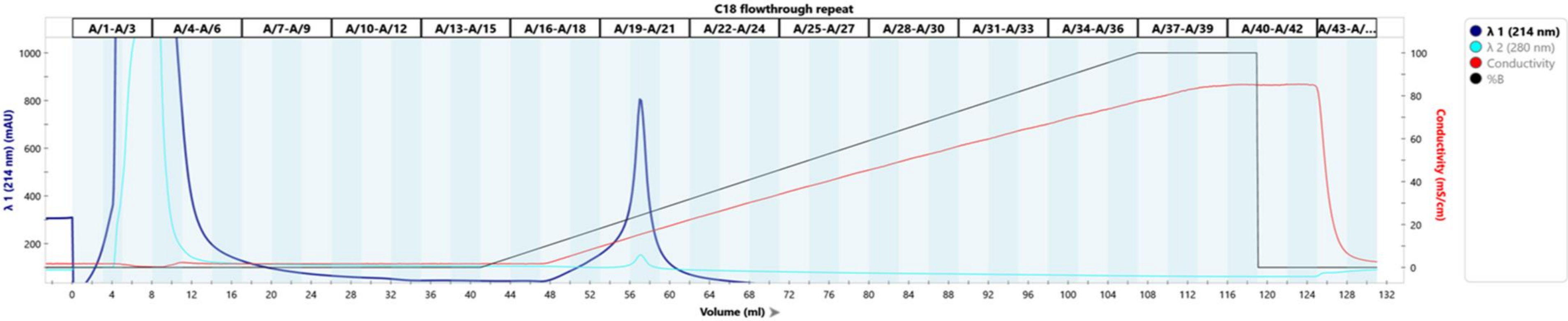

Figure S2. FPLC cation exchange profile showing the purity of plantacyclin B21AG. Eluted protein was detected at 214 nm (blue line) and 280 nm (cyan line).

Table S1. Details of circular bacteriocins used in amino acid sequence alignment and phylogenetic tree generation

Protein name	Reference	Accession number	Mature sequence
Butyrivibriocin AR10	27	AAC69560.1	IADKMGIQLAPAWYQDIVNWVSAGGTLTTGFAIIVGVTVPAWIAEAAAAFGIASAIYF
Acidocin B	28	AJP07153.1	IADQFGIHLATGTARKLLDAVASGASLGTAFAAILGVTLPAWALAAAGALGATAAIYW
Plantacyclin B21AG	31 - 33	WP_105517953.1	IARQFGVHLTTKLTQKALDLLSSGASLGTVAAVILGVTLPGWAVAAAGALGGTAAIVW
Plantaricyclin A	30	WP_053266997.1	IAKQFGVHLTTSLTQKALDLLSAGSSLGTVAAAVLGVTLPAWAVAAAGALGGTAAIVW
Gassericin A	26	WP_012621083.1	IADQFGIHLATGTARKLLDAMASGASLGTAFAAILGVTLPAWALAAAGALGATAAIYW
Paracyclicin	29	ATH00573.1	IYFIANKLGIHLAPGWYQDMVNYVSAGGSLAGAFSVVAGVTLPAWIVPIATAFGAVSA
Amylocyclicin	14	AEK90387.1	LASTLGISTAAAKKAIDIIDAASTIASIISLIGIVTGAGAISYAIVATAKTMIKKYGKKYAAAW
amylocyclicin CMW1	15	AEB64818.1	LASTLGISAAAAKKAIDIIDAASTIASIISLIGIVTGAGAISYAIVATAKTMIKKYGKKYAAAW
AS-48	16	WP_010824081.1	MAKEFGIPAAVAGTVLNVVEAGGWVTTIVSILTAVGSGGLSLLAAAGRESIKAYLKKEIKKKGKRAVIAW
bacA	17	BAA24805.1	MAKEFGIPAAVAGTVLNVVEAGGWVTTIVSILTAVGSGGLSLLAAAGRESIKAYLKKEIKKKGKRAVIAW
Aureocyclicin 4185	12	WP_032495770.1	LTGLGIGTGMAATIINAISVGLSAATILSLISGVASGGAWVLAGAKQALKEGGKKAGIAF
Carnocyclin A	18	B2MVM5.1	LVAYGIAQGTAEKVVSLINAGLTVGSIISILGGVTVGLSGVFTAVKAAIAKQGIKKAIQL
Circularin A	19	WP_077869167.1	VAGALGVQTAAATTIVNVILNAGTLVTVLGIIASIASGGAGTLMTIGWATFKATVQKLAKQSMARAIAY
Enterocin NKR-5-3B	13	2MP8_A	LTANLGISSYAAKKVIDIINTGSAVATIIALVTAVVGGGLITAGIVATAKSLIKKYGAKYAAAW
Garvicin ML	21	WP_003134488.1	LVATGMAAGVAKTIVNAVSAGMDIATALSLFSGAFTAAGGIMALIKKYAQKKLWKQLIAA
lactocyclicin Q	22	BAH29711.1	LIDHLGAPRWAVDTILGAIAVGNLASWVLALVPGPGWAVKAGLATAAAIVKHQGKAAAAAW
Leucocyclicin Q	23	BAL14584.1	LVNQLGISKSLANTILGAIAVGNLASWLLALVPGPGWATKAALATAETIVKHEGKAAAIAW
Uberolysin	24	WP_012657601.1	LAGYTGIASGTAKKVVDAIDKGAAAFVIISIISTVISAGALGAVSASADFIILTVKNYISRNLKAQAVIW
Thermocin 458	20	WP_011230222.1	VAGTLGVSQSIATTVVSIVLTGSTLISIILGITAILSGGVDAILEIGWSAFVATVKKIVAERGKAAAIAW
